# Validity and reliability of the My Jump 2 app for detecting interlimb asymmetry in young female basketball players

**DOI:** 10.3389/fspor.2024.1362646

**Published:** 2024-04-04

**Authors:** Nenad Stojiljković, Dušan Stanković, Vladan Pelemiš, Nebojša Čokorilo, Mihai Olanescu, Miruna Peris, Adrian Suciu, Alin Plesa

**Affiliations:** ^1^ Faculty of Sport and Physical Education, University of Niš, Niš, Serbia; ^2^ Teacher Education Faculty, University of Belgrade, Belgrade, Serbia; ^3^ Faculty of Sport, Union University-Nikola Tesla, Belgrade, Serbia; ^4^ Faculty of Automotive, Mechatronics and Mechanical Engineering, Technical University of Cluj-Napoca, Cluj-Napoca, Romania; ^5^ Faculty Industrial Engineering, Robotics and Production Management, Technical University of Cluj-Napoca, Cluj-Napoca, Romania

**Keywords:** physical performance, mobile application, testing, vertical jump, unilateral, muscle imbalances

## Abstract

**Introduction:**

The aim of this study was to examine the validity and reliability of the My Jump 2 app for the assessment of interlimb jump asymmetry in young female basketball players.

**Methods:**

Nine athletes (age 15 ± 0.9 years; weight 62.9 ± 5.8 kg; height 173.6 ± 6.1 cm) performed single-leg drop jumps (DJs) and both-leg drop jumps on a force plate (Kistler Quattro jump) and were simultaneously recorded on two smartphones using the My Jump 2 app. Jump height from flight time and contact time data were statistically analyzed to evaluate the validity of two different camera settings, drop jump performance, and interlimb jump asymmetry in basketball players. The testing was repeated after 1 week for test retest reliability.

**Results:**

High test-retest reliability [intraclass correlation coefficient (ICC) > 0.88] was observed in DJ height. High correlation between the force plate and the My Jump 2 app was observed in DJ height (*r* = 0.99) and DJ contact time (*r* = 0.98). For the interlimb jump height asymmetries, mean differences were 0.6 percentages for the My Jump 2 app and the force plate, respectively (*p* = 0.77). Inter-device reliability revealed almost perfect correlation for the DJ height (ICC = 0.99, *r* = 0.98).

**Conclusion:**

The My Jump 2 app is a valid and reliable tool to assess drop jump performance and interlimb asymmetry in young female basketball players.

## 1 Introduction

Basketball is a complex team sport that predominantly requires distinctive movement patterns consisting of sprints, change of direction speed, and jumping (1). Moreover, the vertical jump is one of the most common movements during a basketball game (2) but is also very important in a wide range of basketball skills, such as rebounding, blocking, dunking, shooting, and lay-ups. According to Abdelkrim et al. (3), approximately 45 vertical jump acts are performed by elite male basketball players per game. However, vertical jumps in basketball are executed from a single-leg take-off or bilaterally from a standing start (4). Therefore, it is important to understand the type of jumps performed in basketball and what their impact is on both performance and injuries. Accordingly, lower limb asymmetry in basketball can play an important role in addressing the issue of athlete's performance, injury risk, and overall biomechanics. This was confirmed in several studies in which lower limb asymmetry was related to sport performance (5, 6) and injury risk (7–9). In addition, single-leg jump height and reactive strength index are reliable tools to identify lower limb asymmetries in athletes (10). However, new studies are needed to provide more recent results for sport diagnostics and injury prevention or to confirm previous research.

The gold standard for measuring force is isokinetic dynamometers, which can be used for detecting lower limb asymmetry (11). However, an alternative solution that proved to be reliable and valid is the single-leg vertical jump that has also demonstrated good sensitivity to detect lower limb asymmetries (12, 13). Contact mats and force plates are the most used apparatus for measuring and analyzing vertical jump performance in athletes (14, 15). Although today's technology allows us to use more advanced technology for training and science, force plates can be difficult to carry on field testing. Instruments that are more portable than force plates are Optojump photoelectric cells, whose validity and reliability were also confirmed (16). Wheeler jump sensors and infra-based photocell (ADR jumping), both of which are validated, tend to be more portable and economical than Optojump and force plates (17, 18). Another valid way to measure vertical jump performance is by using accelerometers (19). In addition, the My Jump 2 app was created as an easy-to-use and portable mobile application that could precisely measure jump performance (20). The smartphone application My Jump 2 uses the camera to capture slow-motion footage of various jump tasks. By choosing the take-off and landing frame (flight time), it provides us with information regarding jump height (21). Using high-speed cameras, software, and kinematics (MOCAP) are the gold standard for measuring the jump height of athletes (22). However, the portability of the smartphone is unsurpassed. The reliability and validity of the My Jump 2 app were confirmed on the elderly (23), young amateur athletes (24, 25), sport science students (16, 26), trained athletes (27), and even football players with cerebral palsy (28). Specifically, regarding vertical jump asymmetries, Barbalho et al. (29) demonstrated good validity for detecting interlimb contact time and interlimb flight time asymmetry in soccer players using the My Jump 2 app. The primary aim of this research was to test the validity of the My Jump 2 app. The second aim of this study was to examine the use of different camera settings on the My Jump 2 app. Since the application is based on kinematics, our results provide more user-friendly approaches to testing with the My Jump 2 app.

Overall, addressing lower limb asymmetry in basketball can be very important for optimizing performance, reducing injury risk, and maintaining long-term musculoskeletal health. A low percentage of interlimb asymmetry is positively connected to low injury rate and higher performance (30–32). However, studies that aimed to determine the validity and reliability of mobile applications for testing jump asymmetries in female basketball players are lacking. Therefore, the aim of this study was to determine the validity and reliability of the My Jump 2 app for assessing the interlimb jump asymmetry in female basketball players, as well as to confirm the results of previous studies that have carried out research using the My Jump 2 app. It was hypothesized that the My Jump 2 app would show high validity for measuring interlimb asymmetry compared to force plates.

## 2 Methods

### 2.1 Participants

A sample size estimation from a previous study indicated that seven participants were required. An *a priori* power analysis with an effect size of 0.93, a power of 95, and an alpha level of 0.05 was conducted using G*Power (Version 3.1.9.4, University of Dusseldorf, Germany) (27). Nine young female basketball players participated in the study (age 15 ± 0.9 years; weight 62.9 ± 5.8 kg; height 173.6 ± 6.1 cm). The inclusion criteria were as follows: participants had to be included in basketball games and practices for at least 5 years; had been included in any training-related vertical jumps; and had no chronic or acute injury of the lower extremities in the last 6 months. The potential risks, beneﬁts, and discomforts associated with the program were explained to the participants. All participants and their guardians agreed to take part in the study by signing an informed consent form. There is no relationship between the authors and the developers of the application.

### 2.2 Design

Data collection was carried out using smartphones and Kistler Quattro Jump software. Three raters were included in the testing procedure: one controlling force platform and two raters recording with the My Jump 2 app. Testing was done in the afternoon hours. A single session and 1-week separation for the test–retest reliability were performed. Data from the force plate and the My Jump 2 app were recorded simultaneously using two smartphones. Before testing, participants were familiarized with the drop jump (DJ) protocols the day before. In the familiarization phase, both PhD students (former and active athletes) demonstrated the correct technique of performing the drop jump test. Numbers were placed on the participants’ shorts for later recognition and extraction of data. On the day of testing, the athletes performed a regular 10–15 min basketball warm-up (jogging and drills with a ball) with a team and set of static and dynamic stretching exercises. Moreover, a set of five DJs with both legs and five single-leg DJs with submaximal effort were done. After that, athletes were instructed to perform three DJs with both legs and three single-leg DJs. Approximately 1 min of rest was taken between each jump. The athletes were instructed to jump as high as possible. The average of the two attempts and the highest values were analyzed. The athletes did not perform forced dorsal flexion of the ankle during the flight phase. The same procedure was performed after 1 week (in the same shoes and at the same time of day).

Intra- and inter-rater reliability were done analyzing the same video. All data were collected using a 120 Hz camera system, and a 240 Hz camera system was used only for analyzing reliability between two smartphones.

### 2.3 My Jump 2 app

The application was installed on both the iOS operating system (iPhone 11, iOS 16) and the Android system (Xiaomi MI 9 SE, Android 11). The recording was done with a high-speed setting [240 frames per second (fps)/Hz 1,080 pixels and 120 fps/Hz 1,080 pixels, respectively] on both devices. The applications calculated the jump height from the time (flight time) between two frames (measured in ms), representing the loss of foot contact (take-off) with the ground and the recovery of that contact (landing) (26). Version v1.0 of the My Jump 2 app was used. The minimum requirements for the use of the application are Android version 8 and iOS version 12 or higher. This may vary depending on further development of the app.

The contact time and jump height for the left and right legs were analyzed (in ms); contact asymmetry was calculated as the difference between the contact times (contact) of the left and right legs; and jump height asymmetries were calculated as the difference between the flight times of the left and right legs.

For the DJ test, contact time (in ms) and jump height (in cm) data were collected. Two raters recorded all jumps simultaneously (two phones). The raters and smartphones were positioned in the frontal plane, 1.5 m from the athlete in a seated position side by side. The camera position was set at the patella (kneecap) height of the participant standing on the force plate. The device positions were set without a tripod setting for more realistic simulation and practical use of the My Jump 2 app.

### 2.4 Force platform

The Quattro Jump 9290BA force plate (Kistler, Winterthur, Switzerland) was used (33). Data were recorded at a sampling frequency of 500 Hz. The force plate calculated the same variables as the My Jump 2 app (jump height and contact time). Time between the take-off and landing phases was used to establish jump heights and contact times.

### 2.5 DJ assessment

**Figure 1:**
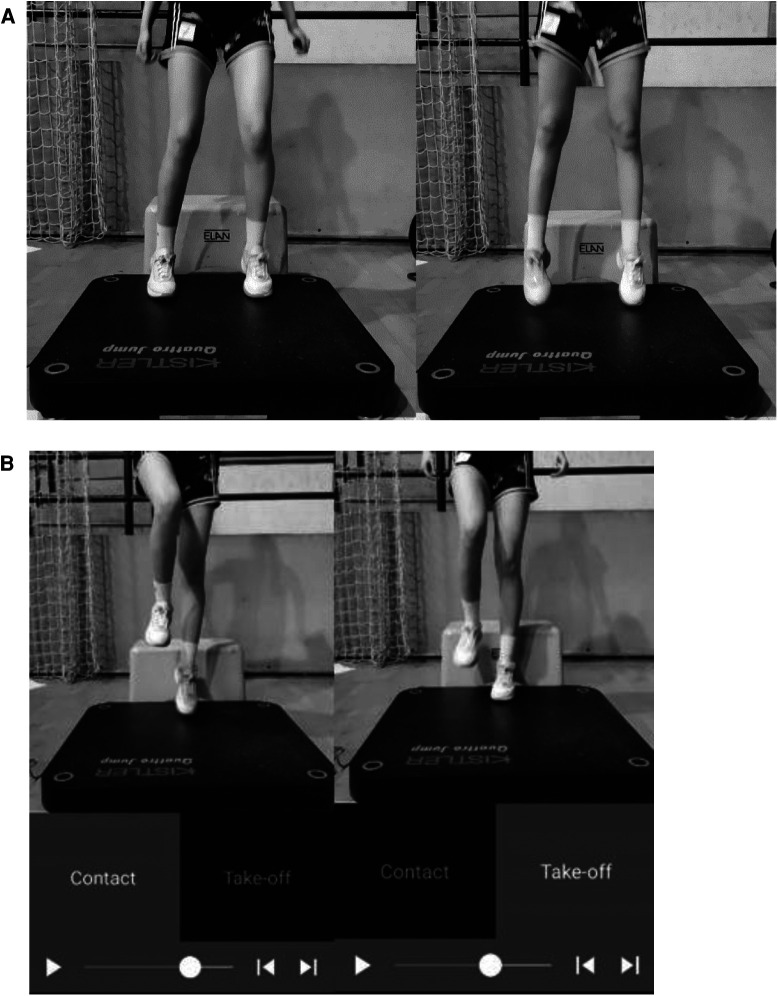
Drop jump (**A**) and single-leg drop jump (**B**).

The athletes started the DJ test in a normal upright position standing on a box height of 30 cm. This height was agreeable with the previous study suggesting drop heights near 30 cm (34) in this age and sex range (35). The rater who was assessing the force plate measurements signaled twice. The first “start” command was for the two raters recording with an app, and the second “start” was for the athlete to perform the DJ. The athlete stepped toward a force plate that was set up in front of the box, moving with both feet. The participant immediately performed a squat with a preferred knee angle. The athlete then extended their knees to jump as high as they could while pushing their body vertically (Figure 1A).

### 2.6 Single-leg DJ

Figure 1B displays the single-leg DJ for the left lower limb. The box height was set at 30 cm. The rater signaled twice, once for the two other raters and once for the athlete.

The athlete stepped in front of a force plate that was set up in front of the box. A single-leg jump with a preferred knee angle was performed by the participant as soon as the foot stepped in contact with the plate. The athlete then extended her knee to jump as high as she could while pushing her body vertically. This test was performed three times, alternating both legs.

### 2.7 Statistical analysis

Means and standard deviations were used as descriptive statistics. The Shapiro–Wilk test was used to check the data normality due to the small sample size. The asymmetry in contact time and jump height metrics were used to compare the mean differences between the devices using paired samples *t*-tests. A single measure, two-way mixed, absolute agreement parameter was chosen for the intraclass correlation coefficient (ICC) analysis for intra-rater reliability and average measures, two-way mixed, absolute agreement parameter was selected for the inter-rater reliability. An interpretation of the ICC was given as follows: <0.5 = poor reliability; 0.5–0.75 = moderate reliability; 0.75–0.9 = good reliability; and >0.90 = excellent reliability (34). Reliability between the test–retest was analyzed using the ICC. Pearson's product-moment correlation was used to test the concurrent validity of the application. In addition, Bland–Altman plots were used to visually analyze the agreement between the My Jump 2 app and force plate data. Bland–Altman plots showed the difference between the two devices plotted against the mean of the two devices. Typical error (TE) expressed as the coefficient of variation (CV%) was used in reliability measures (CV < 5% = good reliability) (36). Mean absolute percentage error (MAPE) was used for validity measures (<10% = highly accurate, >10% = good; >20% = reasonable) (37).

## 3 Results

Table 1 presents the inter- and intra-rater reliability in the drop jump test, with almost perfect values of consistency.

**Table 1:** ICC of inter- and intra-rater reliability.

Variable	Rater 1 vs. Rater 2 (ICC, 95% CI)	Rater 1a vs. Rater 1b (ICC, 95% CI)
Drop jump		
Jump height (cm)	1	1
Contact time (ms)	0.998 (0.992–1)	1
Single-leg jump		
Jump height right (cm)	0.980 (0.90–0.99)	0.995 (0.979–999)
Contact time right (ms)	0.994 (0.975–0.999)	0.998 (0.993–1)
Jump height left (cm)	1	1
Contact time left (ms)	0.997 (0.987–0.999)	1

The My Jump 2 app showed a high validity with the force platform. For the interlimb asymmetry test, there were no significant differences in any of the variables for the interlimb asymmetry test between the force plate and the My Jump 2 app (Table 2). For the drop jump test, there was no significant difference in jump height variable (*p* = 0.97) and the results showed a very large correlation for DJ jump height values between the two devices (ICC = 0.99; *r* = 0.99). The contact time for the drop jump test showed the largest mean difference between the application and force plate (367 vs. 385 ms, *p* = 0.7) (Table 2). The highest values are presented in Table 2.

**Table 2:** ICC and mean difference of My Jump 2 app and force plate.

Variable	My Jump 2	Force plate	MAPE	CV% (CI%)	*p*	MD	ICC (CI%)	*r*
Interlimb asymmetry								
Jump height asymmetry (%)	5.7	6.3	12.9%		0.8	0.6	0.96 (0.8–0.99)	0.93
Contact time asymmetry (%)	7.9	5.72	24.6%		0.5	2.3	0.94 (0.72–0.99)	0.90
Jump height right (cm)	12.9 (3.6)	13.2 (3.2)	6.7%		0.9	0.3	0.98 (0.91–0.99)	0.97
Contact time right (ms)	414 (104)	426 (119)	4.9%		0.8	12.1	0.98 (0.9–0.99)	0.96
Jump height left (cm)	12.79 (3.4)	13.01 (3.7)	3.7%		0.8	0.45	0.99 (0.93–0.99)	0.98
Contact time left (ms)	458 (111)	448 (113)	3.1%		0.9	9.63	0.99 (0.95–0.99)	0.99
Drop jump								
Jump height (cm)	24.82 (4.6)	24.9 (5.1)	2.6%		0.9	0.08	0.99 (0.97–0.99)	0.99
Contact time (ms)	367 (77)	385 (118)	4.8%		0.7	18.6	0.92 (0.28–0.98)	0.98
Test–retest								
	My Jump 2 Test	My Jump 2 Retest						
Drop jump (cm)	23.7 (6.5)	24.8 (4.6)		1.9 (1.4–2.3)	0.7	1.16	0.88 (0.74–0.98)	0.95
Contact time (ms)	367.3 (106)	366.9 (77)			0.9	9.96	0.6 (−0.95–0.92)	0.45
Inter-device reliability								
	120 fps	240 fps						
Drop jump (cm)	24.8 (4.6)	24.2 (4.9)		0.7 (0.5–0.8)	0.8	0.6	0.99 (0.93–0.99)	0.98
Contact time (ms)	367 (77)	357 (76)			0.8	9.9	0.93 (0.72–0.99)	0.87

Data are mean (SD).

MD, mean difference; *r*, Pearson's product-moment correlation coefficient.

Test–retest showed good reliability for jump height in the drop jump test (ICC = 0.88) (Table 3) and poor to moderate reliability for contact time (ICC = 0.46) (Table 3). Different camera settings showed excellent reliability for jump height (ICC = 0.99) and excellent reliability for contact time (ICC = 0.93). The MAPE for single-leg outcomes and for drop jumps with both legs showed a percentage error below 10%, indicating good results (Table 2). Moreover, jump height asymmetry and contact time asymmetry showed fair to poor MAPE results (>10%). The average of the two attempts measured using the My Jump 2 app and the force plate are presented in Table 3.

**Table 3:** Mean ± SD for jump heights and contact time recorded with My Jump 2 app and force plate.

	My Jump 2	Force plate	*r*
Jump height right (1) (cm)	11.9 (3.2)	12.3 (3.3)	0.99
Contact time right (1) (ms)	409 (102)	420 (113)	0.96
Jump height right (2) (cm)	12.1 (3.1	12.5 (3.3)	0.97
Contact time right (2) (ms)	417 (88)	431 (79)	0.90
Jump height left (1) (cm)	11.2 (3.2)	11.6 (3.4)	0.98
Contact time left (1) (cm)	396 (78)	410 (96)	0.94
Jump height left (2) (cm)	12.5 (3.4)	12.7 (3.6)	0.99
Contact time left (2) (cm)	409 (82)	423 (76)	0.95
Jump height both legs (1) (cm)	21.6 (5.1)	22.1 (5.4)	0.99
Contact time both legs (1) (ms)	370 (78)	392 (84)	0.96
Jump height both legs (2) (cm)	22.5 (6)	22.9 (5.7)	0.99
Contact time both legs (2) (ms)	343 (77)	362 (74)	0.90
	120 fps	240 fps	
Drop jump (1) (cm)	23.5 (4.6)	23.3 (4.8)	0.99
Contact time (1) (cm)	379 (74)	373 (76)	0.86
Drop jump (2) (cm)	24.2 (4.6)	23.7 (4.7)	0.99
Contact time (2) (cm)	389 (77)	377 (79)	0.86

*r*, Pearson's product-moment correlation coefficient.

**Figure 2:**
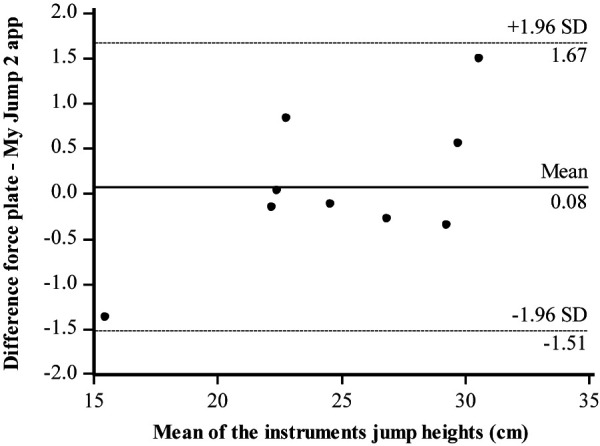
Level of agreement (Bland–Altman) with 95% limits of agreement (dashed lines) and the mean difference (solid line) between force plate and My Jump 2 app for DJ with both legs.

**Figure 3:**
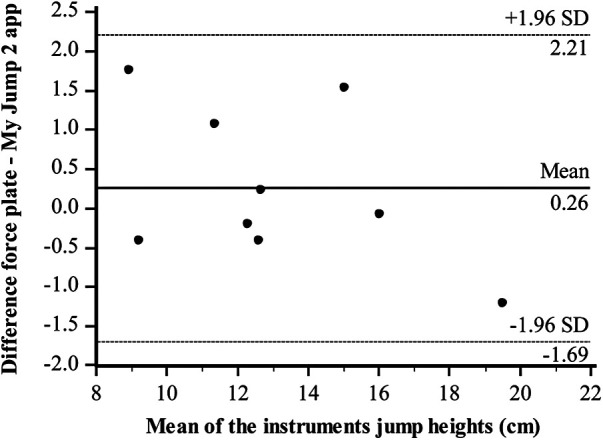
Level of agreement (Bland–Altman) with 95% limits of agreement (dotted lines) and the mean difference (solid line) between Force plate and My Jump 2 app in the jump height (right leg).

**Figure 4:**
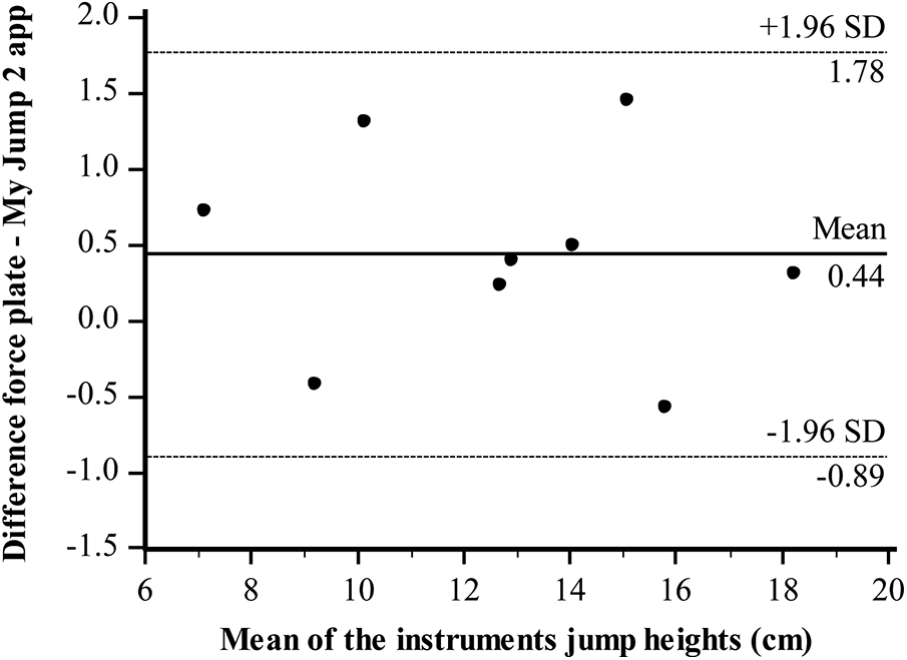
Level of agreement (Bland–Altman) with 95% limits of agreement (dotted lines) and the mean difference (solid line) between force plate and My Jump 2 app in the jump height (left leg).

Bland–Altman plots regarding the jump height of both-leg DJs were illustrated between the two instruments. The Bland–Altman difference plot represents the mean of the two instruments (cm) in the *x*-axis and the difference between the force plate and My Jump 2 app in the *y*-axis. All the data points were within the 95% confidence intervals (CIs). Bland–Altman plots showing the limits of agreement for DJs between the force plate and the My Jump 2 app (Figure 2), for the right leg (Figure 3) and left leg (Figure 4), are in good agreement. There is no apparent systematic bias, as the differences are randomly scattered above and below the mean difference line. The majority of data points cluster around the zero-difference line, suggesting that the measurements from the My Jump 2 app and the force plate are in close agreement with each other. This indicates that both methods effectively measure jump height.

## 4 Discussion

The purpose of this study was to examine the reliability and validity of the My Jump 2 app between one and two raters, between different camera settings, and interlimb asymmetry in female basketball players. The findings confirmed high validity and reliability in testing the jump height from flight time and contact time compared to a force platform. Moreover, different frames per second in video recording with the My Jump 2 app showed a high ICC. To the best of the authors’ knowledge, no study has compared the force plate (Kistler Quattro Jump) and My Jump 2 app with young female basketball players.

Interlimb asymmetry has been identified as an essential factor in jumping performance in youth team-sport athletes (38). In fact, female basketball players who compete in higher leagues tend to have lower interlimb jump height asymmetry (39). Therefore, strengthening the weaker leg and reducing interlimb asymmetry can improve athlete's overall jumping performance (37). Our findings are consistent with previous results (29), showing high validity when comparing the My Jump 2 app and force plate in soccer players (*p* = 0.91 and *p* = 0.88) for flight time and contact time, respectively. Although there are similarities with previous research, MAPE statistics were not observed (29) and cannot be compared. In addition, results from our study showed high validity but are limited for MAPE for both jump height and contact time asymmetry (>10%). However, our findings indicate high ICC and Pearson correlation values (>0.90) for recognition of the interlimb asymmetry using the My Jump 2 app compared to the force plate in the young female basketball population. This is very important, considering coaches can evaluate lower limb asymmetries with an easy-to-use and portable device. Moreover, if it is used with a young population as a monitoring tool, it can prevent early career injuries (40).

Drop jump exercises have been proven to have a major role in enhancing explosive power in basketball players (41). The present data showed a strong correlation in DJ between the force platform and the My Jump 2 app (*r* = 0.99) for jump height (*r* = 0.98) and for contact time (MAPE <5%). Although our outcomes showed a high correlation for contact time, *p*-values were marginally lower for contact time asymmetry (%) and DJ contact time (ms). Our results showed similar outcomes compared to Gallardo-Fuentes et al. (27), who found a significant correlation in DJ height (*r* = 0.99) between the two already mentioned instruments. Another study identifies excellent ICC values for DJs (29) (0.97 and 0.94) for jump height and contact time, respectively. These results can be compared with ours (0.99 and 0.92) for jump height and contact time in DJs. These results can be explained by already developed applications and multiple studies that assessed the My Jump 2 app with various vertical jump tests. Comyns et al. (19) provided evidence of excellent reliability between force plates, photoelectric cells, and accelerometers but only the countermovement jump variable, drop jump, and asymmetry test were not investigated. Adding kinematics methods, such as the My Jump 2 app, for comparison in addition to these three aforementioned methods could be interesting for future findings. This could provide more proof for the reliability and diversity of instruments that can be used for measuring interlimb asymmetries and performance in athletes.

The test–retest method has shown to be an important method for calculating the reliability of a test (42–44). In this study, test–retest results in DJs in young female basketball players revealed good to excellent reliability in jump height outcomes (ICC = 0.88) and poor to moderate contact time outcomes (ICC = 0.460). The current results for jump height are in accordance with those of a previous study, which examined countermovement jumps and squat jumps instead of DJs (45). Another study (27) confirmed that the My Jump 2 app has high test–retest values for countermovement jumps, squat jumps, and DJs (*r* = 0.86–0.95). Therefore, the My Jump 2 app was shown to be reliable for measuring jump height in athletes.

Intra- and inter-rater reliability have an important role in evaluating the consistency of an instrument in one and two assessors (46). Our findings can be compared to the results of earlier studies (29, 47) that evaluated the intra- and inter-rater reliability of the My Jump 2 app. The present results for intra- and inter-rater reliability show perfect and almost perfect correlations for all the variables tested (ICC > 0.99). Rogers et al. (47) evaluated the inter-rater reliability of the My Jump 2 app with an almost perfect correlation performing a countermovement jump (cm) (ICC = 0.99). The results of raters assessing the reliability of the My Jump 2 app can be found in the study by Barbalho et al. (29). Moreover, in their study, the intra- and inter-rater reliability yielded perfect and almost perfect correlations (ICC > 0.99 and ICC > 0.98, respectively) for single-leg DJ contact time and both-leg DJ jump height and contact time, consistent with our findings. These findings can justify the usage of mobile applications with both different raters and a single rater. It is important that the device can be used in different situations since the My Jump 2 app is mostly used in field-testing conditions and it is important that any rater can perform the testing.

Higher fps in cameras allow for more precise video analysis (48). This is because a higher fps captures more frames of movement, which can be used to track and analyze the motion of objects in the video more accurately. In addition, our study provides evidence of ICC results using two different frame settings (120 and 240 fps). To date, no study has looked specifically at the correlation between two models of smartphones and different frame rates when using the My Jump 2 app. Haynes et al. (20) discussed that the frame rate can be a limited factor in the accuracy of the My Jump 2 app. Our data indicate excellent reliability for DJ jump height (ICC = 0.99) and excellent reliability for DJ contact time (ICC = 0.93) comparing these two settings. The results provide evidence that the quality of the device is not crucial for the accuracy of the measurement in the My Jump 2 app, only the frame rate of a video, which can be either 120 or 240 fps. These findings will make the My Jump 2 app more accessible to a wider range of users.

Although we statistically managed to group the required sample size, more participants could increase the statistical power and representativeness of the study. Our sample size estimation indicated a requirement for seven participants. However, the *p*-values for jump height and contact time asymmetries (%) were 0.77 and 0.52, respectively. A bigger sample size may show larger differences. Furthermore, a small sample size may not provide sufficient statistical power to detect significant relationships of validity and reliability of the My Jump 2 app. Second, participants are young female basketball players who find it difficult to perform single-leg DJs due to their anthropometric characteristics, age, and technique. Our study may have been limited by the fact that some participants may not have been familiar with the single-leg DJ technique. Even though the technique was demonstrated the day before testing and participants had a familiarization phase, it was not sufficient time to learn the proper technique of the single-leg drop jump. All video recordings were done in a slow-motion setting as suggested by the My Jump 2 app guidelines. Previous research that investigated the My Jump 2 app used 120 Hz or more for data analysis. Lowering the frames per second in the camera settings for comparison analysis may yield reliable results, even when using more affordable devices.

The high correlation between the My Jump 2 app and the force plate in asymmetry tests shows strong evidence for the reliability of a low-cost app. In addition to the asymmetry variable, vertical jump performance can also be measured with high reliability. Moreover, our findings suggest that any rater with or without experience in sport science testing and minimal equipment can measure vertical jump performance with high validity and reliability. These results should encourage sport practitioners to use the My Jump 2 app to a greater extent for enhancing the overall performance of an athlete and reduce injury risk.

## 5 Conclusion

The My Jump 2 app is a reliable and valid instrument for assessing interlimb asymmetry, jump height, contact time, and DJ performance in young female basketball players. In addition, different camera settings proved to be reliable for assessing DJ performance. Therefore, the My Jump 2 app is a low-cost and easy-to-use instrument that basketball coaches can use to improve, monitor, and enhance female basketball players jumping performance.

## Scope statement

This study investigates the validity and reliability of a mobile app designed to detect interlimb asymmetry in female basketball players. The app utilizes video analysis to assess asymmetry in key performance metrics (jump height, contact time) and aims to provide a practical tool for coaches and trainers. We compare the My Jump 2 app measurements against a force plate T (Kistler Quattro Jump) to assess concurrent validity. Moreover, we evaluate the app's intra- and inter-rater reliability and test–retest reliability through repeated measurements.

Interlimb asymmetry can impact performance and increase injury risk, especially in young female athletes. This study addresses the urgent need for reliable and accessible tools for its detection in female basketball athletes. The app’s potential benefits include: Injury prevention (early identification of asymmetry can prompt corrective measures to prevent overuse injuries); Performance optimization (coaches can tailor training programs to address individual asymmetries and improve overall performance); and Accessibility (the app offers a cost-effective and convenient tool for coaches and players compared to traditional methods).

This research fills a critical gap in sports technology and aligns with the journal’s focus on innovative tools for coaching female athletes and injury prevention. We believe our findings will be of significant interest to coaches, trainers, and researchers dedicated to optimizing the performance and health of female basketball players.

## Data Availability

The original contributions presented in the study are included in the article/Supplementary Material, further inquiries can be directed to the corresponding author.
